# Cellular angiofibroma in the retroperitoneal space during pregnancy: A case report

**DOI:** 10.1186/s12905-023-02182-w

**Published:** 2023-02-10

**Authors:** Hanae Abe, Sari Nakao, Hiroya Itagaki, Yoshihiko Hosokawa, Ayumi Shikama, Nobutaka Tasaka, Azusa Akiyama, Takeo Minaguchi, Toyomi Satoh

**Affiliations:** Department of Obstetrics and Gynecology, Faculty of Medicine, University of Tsukuba, 1-1-1 Tennoudai, Tsukuba, Ibaraki 305-8575 Japan

**Keywords:** Cellular angiofibroma, Aggressive angiomyxoma, Angiomyofibroblastoma, Pregnancy, Intraperitoneal space

## Abstract

**Background:**

Cellular angiofibroma (CA) is a rare, benign mesenchymal tumor first described by Nucci et al. (Am J Surg Pathol 21:636–644, 1997. 10.1097/00000478-199706000-00002). It affects both men and women, although it is more common in middle-aged women. CA is well circumscribed and usually observed on the body surface, primarily in distal genital regions. Aggressive angiomyxoma and angiomyofibroblastoma are clinically and histologically similar; therefore, it may be necessary to distinguish between CA and these similar tumors. We present a rare case of CA, with atypical features, in the retroperitoneal space during pregnancy.

**Case presentation:**

The presence of a 130 mm tumor was detected in a 19-year-old woman. The tumor, located in the retroperitoneal space, was found during first pregnancy examination. At 16 weeks of gestation, the woman developed nausea and fever, and it was diagnosed with acute pyelonephritis. After a few days, the amniotic membranes prematurely ruptured, leading to a miscarriage. The woman underwent a tumor resection, after miscarriage. This case presented with atypical features of CA. This included the young age of the patient, and presence of a tumor in the retroperitoneal space.

**Conclusion:**

In this case, the diagnosis of CA was difficult due to the rarity of the disease and its atypical clinical features. From this experience, we recommend that the discussion on the efficacy of surgical treatment and pregnancy outcomes should be done based on individual case, and not generalized.

## Background

Cellular angiofibroma (CA), a rare and frequently asymptomatic benign mesenchymal neoplasm, was first described in a case study by Nucci et al. [1]. It occurs in both sexes and mostly affects women in their fifties. This tumor contains spindle cells and exhibits vascular hyperplasia. It is generally small, has well-circumscribed margins, and arises in the vulvovaginal region. Aggressive angiomyxoma (AA) and angiomyofibroblastoma (AMFB), similar non-epithelial benign tumors, are differential diagnoses of CA, and they affect women of reproductive age. CA seldom affects other sites, apart from the vulvovaginal region. A previous study reported an occurrence of CA in the upper (left axilla) or lower (knee lateral) limbs and trunk [2]. This case study reports a rare, clinically atypical case of CA that was diagnosed during pregnancy.

## Case presentation

**Fig. 1:**
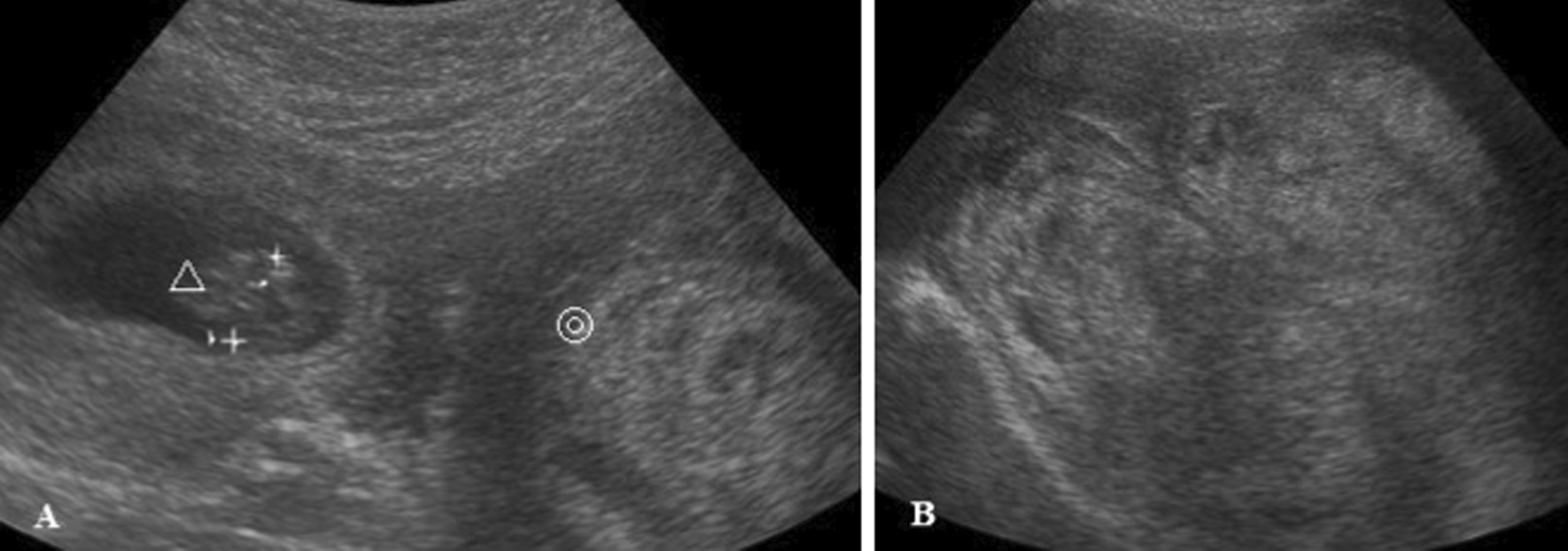
Transabdominal ultrasonography at 9 weeks of gestation. △ Fetus, ◎ Tumor. **A** The uterine body was shifted superiorly by the tumor. **B** Tumor size was 130 mm. It had a hyperechoic region, mainly with a partially low region

**Fig. 2:**
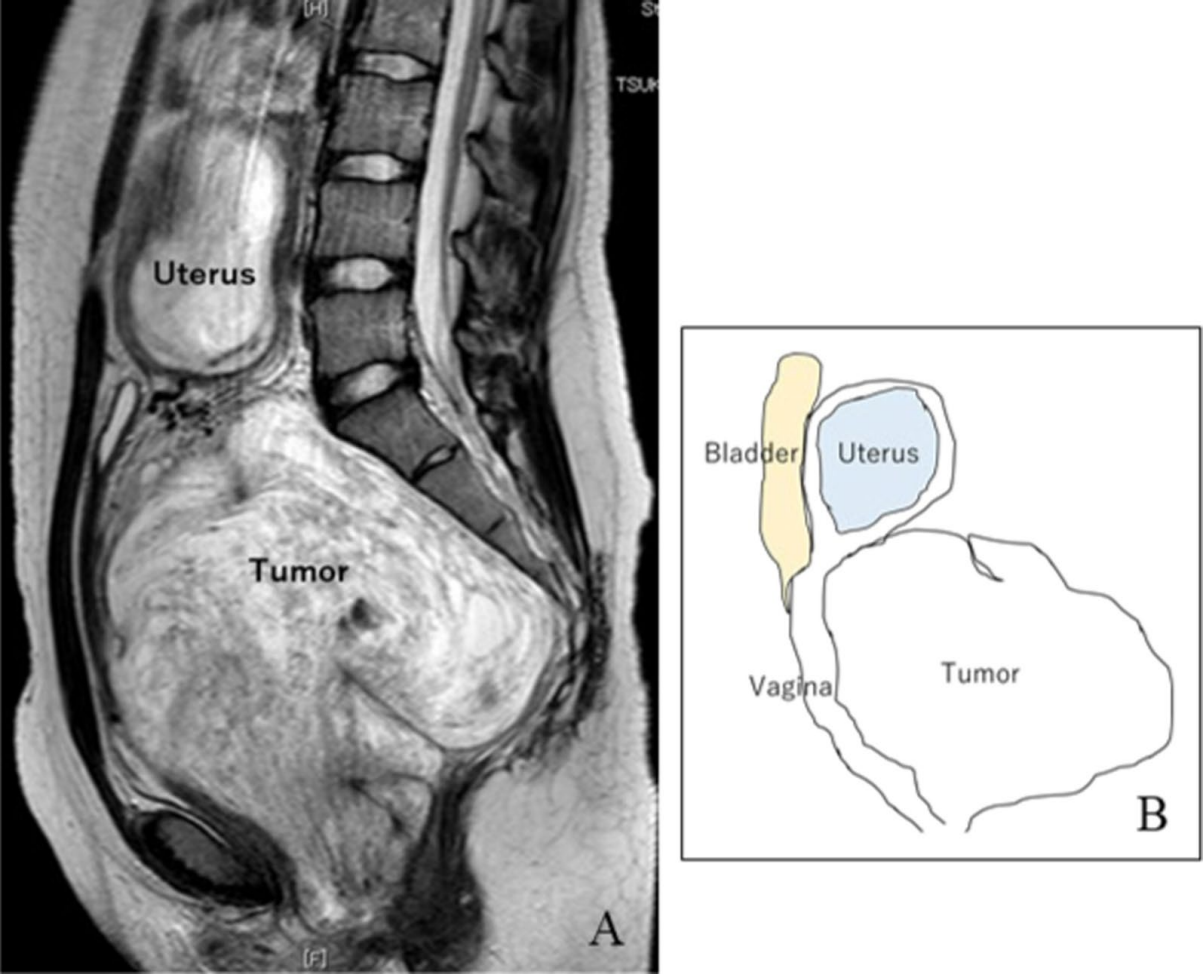
Initial magnetic resonance imaging (MRI) findings. **A** MRI at 12 weeks of gestation. The tumor measured 115 mm × 150 mm × 160 mm. It showed high signal intensity bands, such as whorl-like formation on T2-weighted images. **B** Schematic illustration of MRI scan

A 19-year-old woman with no significant history or symptoms of CA tumor (gravida 1, para 0) underwent her first pregnancy examination at 8 weeks of gestation. She was found to have a giant pelvic tumor at a hospital where she initially consulted. She was referred to our institution for tumor management. In our institution, initial examination revealed a pelvic tumor measuring 130 mm. The fetus was not easily detected using transvaginal ultrasonography; hence, it was detected using transabdominal ultrasonography (Fig. 1). Notably, the levels of tumor makers, including CA125, CA19-9, CEA, SCC, NSE, and LDH, were all within the normal range. Magnetic resonance imaging (MRI) was performed at 12 weeks of gestation (Fig. 2) to evaluate the tumor, which measured 115 mm × 150 mm × 160 mm. Moreover, MRI revealed a bulky, well-circumscribed mass in the retroperitoneal space, with low- and high-signal-intensity bands on T1- and T2-weighted images, respectively. There were significant high-signal-intensity bands on diffusion-weighted MRI, and the tumor was assumed to have a strong swelling lesion. There were whorl-like formations within the tumor, which included a fat component. These imaging findings led to a suspicion of AA, with differential diagnoses of CA and AMFB.

The potential complications and management options were explained to the patient, and she initially chose to continue the pregnancy, since AA is a benign tumor.

At 16 weeks of gestation, the patient developed nausea and fever. The examination doctor suspected ileus because of the tumor and, after a comprehensive examination, diagnosed the patient with gastroenteritis. Nevertheless, the following day, she experienced persistent lower abdominal pain; thus, she was re-examined and found to have abdominal and costovertebral angle tenderness, without signs of peritoneal irritation. Laboratory analysis revealed increased white blood cell count (18,800/μL), elevated C-reactive protein level (8.36 mg/dL), and pyuria. There were no signs of hydronephrosis, and the patient was diagnosed with acute pyelonephritis based on the above-mentioned findings. Consequently, she was administered antibiotics (ceftriaxone [CTRX] [1 g/day]). Three days after admission, the amniotic membranes prematurely ruptured, leading to a miscarriage. The pathological diagnosis of the placenta was stage II of chorioamnionitis [3]. Six weeks after the miscarriage, the patient underwent surgical tumor resection.

MRI was performed 4 weeks after miscarriage to re-evaluate the tumor size and location as well as to screen for malignant transformation. Although the pelvic tumor with hypervascularity had not grown, tumor infiltration into the levator ani muscle was suspected.

**Fig. 3:**
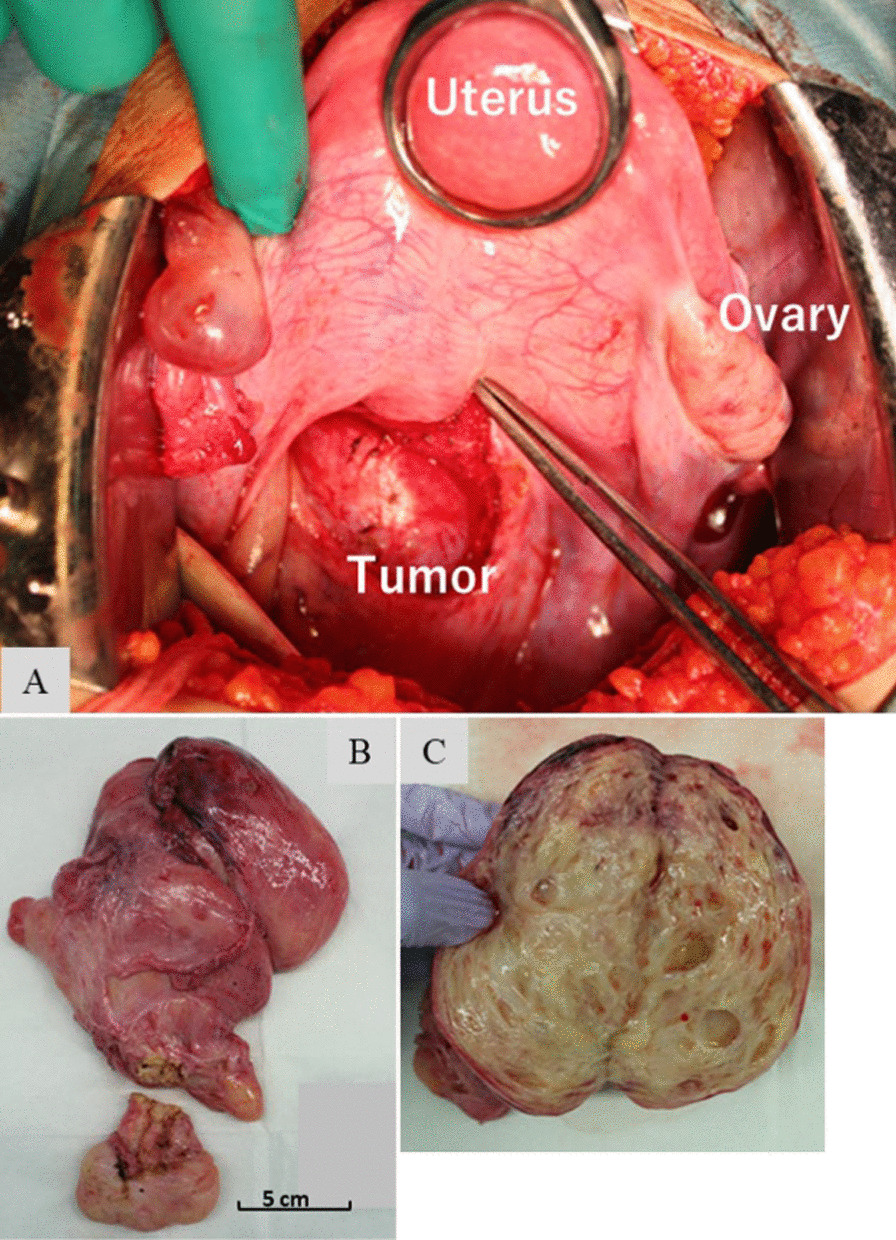
Intraoperative findings and gross appearance. **A** The tumor grew caudally in the retroperitoneal space and occupied the posterior uterine to the rectovaginal regions. **B** Tumor size is 170 × 138 × 70 mm. **C** The tumor is yellowish and has multiple small cysts

**Fig. 4:**
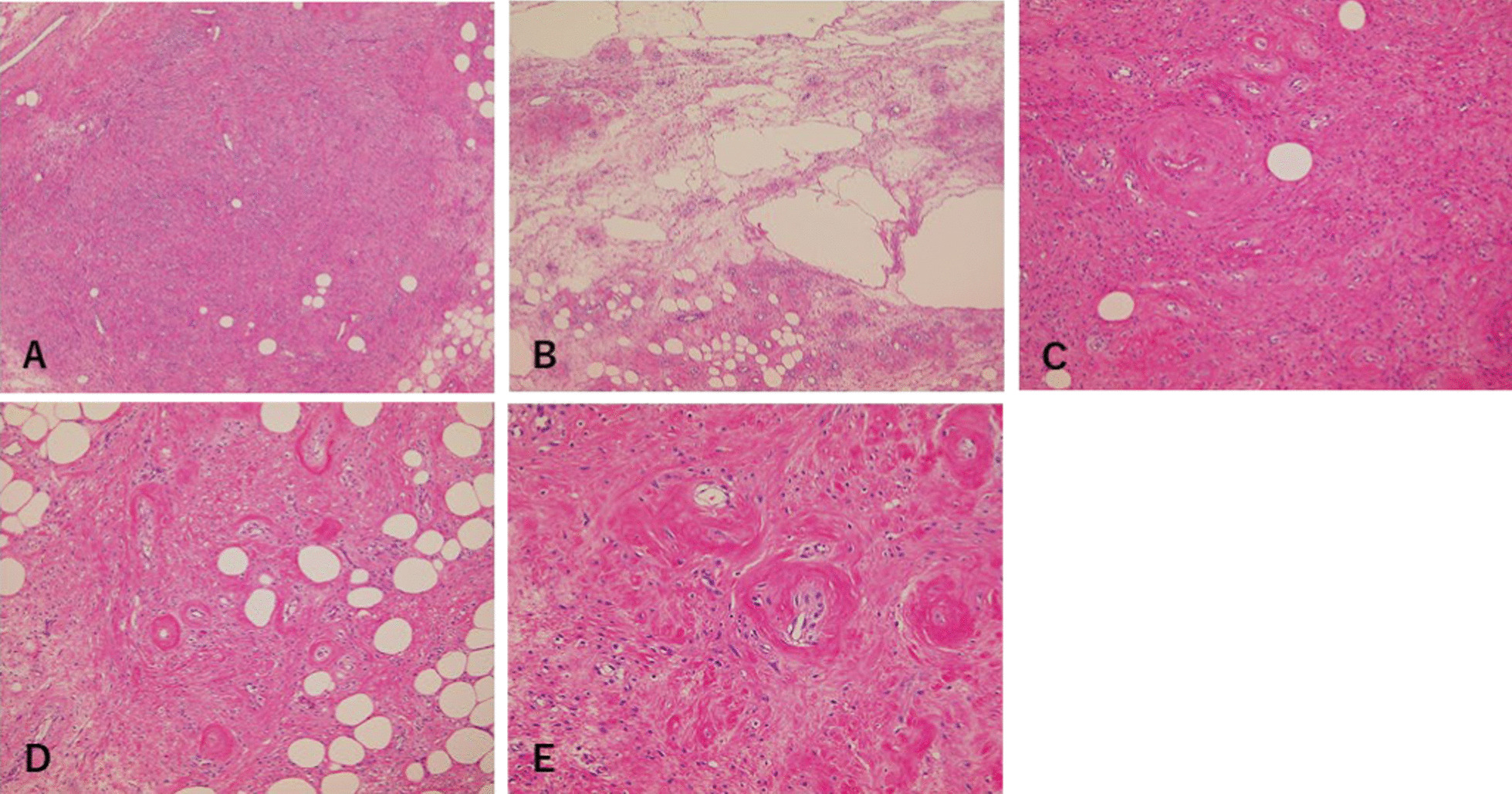
Hematoxylin and eosin staining. No atypical cells or necrosis was observed. **A** (magnification ×40): Adipose cells and slight vascular hyperplasia. **B** (magnification ×40): Constituting the cyst with myxomatous change edematous liquefaction. **C** (magnification ×100): Spindle-shaped tumor cells were transferred from the blood vessel to the interstitial tissue. **D** (magnification ×100), **E** (magnification ×200): Vessel walls, which were small- to medium-sized, showed hyalinization

**Fig. 5:**
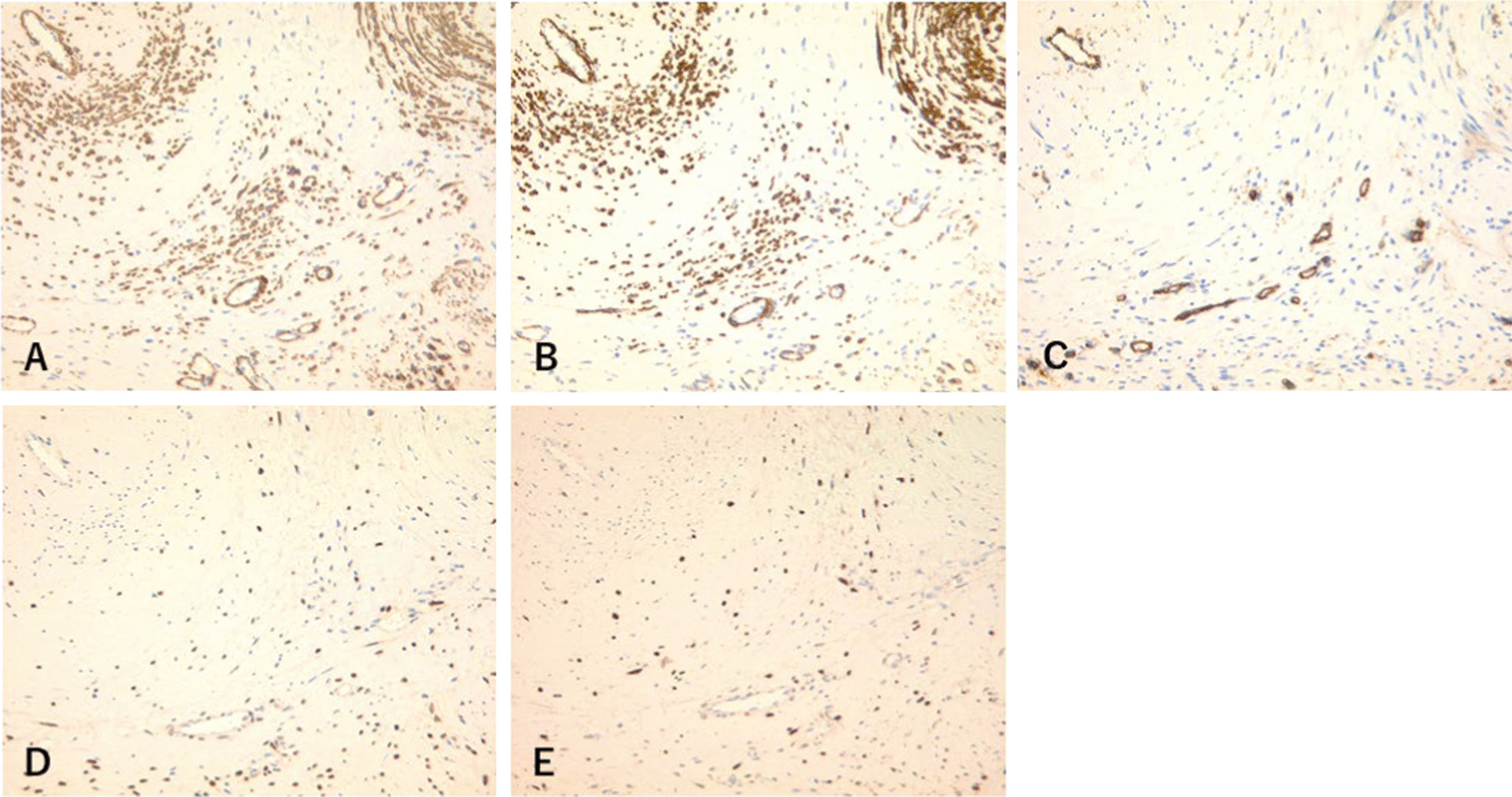
Immunohistochemical staining. **A** αSMA and **B** h-caldesmon are partially positive in the tumor cells, except for the blood vessels. **C** CD34 is partially positive in spindle cells. **D** ER and **E** PgR are positive in most tumor cells

Tumor resection was performed following bilateral ureteral stent placement to prevent ureteral injury. Caudally, the tumor was found in the retroperitoneal space and occupied part of the posterior uterine and rectovaginal regions (Fig. 3). Part of the tumor surface adhered to adjacent structures, and no invasion was observed. A small part of the vagina was ruptured during resection due to the closeness of the surface of the tumor to the vagina, and a transvaginal approach was required. Spindle cells proliferated from blood vessels to interstitial tissues, and the vessel walls were hyalinized. No atypical cells or necrosis was observed (Fig. 4). Immunohistochemical staining of the tumor cells was diffusely positive for vimentin. Most of the tumor tissues, except for blood vessels, was α smooth muscle actin (αSMA)- and h-caldesmon-positive, and spindle cells were slightly CD34-positive. Furthermore, most tumor cells were estrogen receptor (ER)- and progesterone receptor (PgR)-positive. Based on these results, CA, AA, and AMFB as differential diagnoses were distinguished (Fig. 5).

The tumor samples were sent to another institution for pathological diagnosis, and the histopathologic findings were consistent with those obtained at our institution. Meanwhile, immunohistochemical staining of the tumor cells was positive for αSMA, ER, and PgR, and slightly positive for h-caldesmon, CD43, and CD99. However, the staining was negative for S-100. Neither HMGA2 nor RB1 expression was detected. The patient was diagnosed with CA based on the above-mentioned findings, although the clinical findings were not typical.

Postoperatively, on day 3, the patient developed a hematoma (3 cm) in the rectovaginal space, which grew to a maximum size of approximately 10 cm by the 13th day. A drain was inserted transvaginally into the rectovaginal space for hematoma drainage. On postoperative day 29, the hematoma volume reduced significantly (3.5 cm), and the patient was discharged. MRI was performed 3 months postoperatively, no residual tumor was found, and the uterine position was normal. Postoperative follow-up was conducted for up to 5 months, although the patient was lost to follow-up after this period.

## Discussion and conclusions

Several studies reported that CA has a small size, arises in the vulvovaginal area, occurs close to the body surface, and affects quinquagenarian women [1, 2, 4–7]. This study describes a difficult-to-diagnose case of CA with atypical clinical characteristics, in a pregnant woman. The patient in this study was incidentally diagnosed with a pelvic tumor during her pregnancy examination. The patient chose the continuation of pregnancy based on these reasons: (1) the tumor was considered benign, (2) it was suspected to have invaded adjacent organs and anal function could be lost if surgery was done. However, the patient had pyelonephritis, which may have led to the miscarriage. Nevertheless, we could not rule out the possible effect of this giant tumor on the pregnancy loss.

Most patients with CA, a benign mesenchymal neoplasm with smooth margins and relatively small size, are asymptomatic. CA growth occurs over a number of years and commonly affects the vulvovaginal region. According to previous reports, CA is often clinically diagnosed as a Bartholin's cyst [2, 8, 9]. Mandota et al. reported that the mean age of all 79 patients with CA in their study was 46 years with the most common site being the vulvovaginal region. Moreover, the mean and maximum sizes were 3.6 cm and 12.3 cm, respectively [2].

CA is made of spindle cells and thin-walled vessels of various sizes, with the presence of hyalinization. For immunohistochemical examination, in the past reports, the expression of vimentin was positive, and ER and PR were mostly positive. Previous studies have reported different findings regarding the expression of CD34 and desmin [2, 4, 5, 10]. In 2016, Khmou et al. reported that patients with CA showed a loss of the *RB1* gene due to a deletion of the 13q14 chromosomal region, although this is not a routine finding in these patients [4]. In contrast, Chein et al. reported that the two CA patients in their study had a deletion of the *RB1* gene.

Furthermore, six patients with CA having atypia and sarcomatous transformation had a deletion of *RB1*. Based on these results, the deletion of the *RB1* gene may be considered as one of the parameters of atypia or sarcomatous transformation in patients with CA [11]. CA should be distinguished from other differential diagnoses [2, 4, 12–15]. Tumor features such as tumor morphology, cytological features, and immunohistochemical findings can overlap with those of other tumors; thus, all the above-mentioned features should be comprehensively considered when making a diagnosis of CA.

In this case study, it was necessary to distinguish between CA, AA, and AMFB. Therefore, another institution was consulted, and no HMGA2 expression in the cell nucleus and a reduced or no *RB1* expression were found [4, 11]. The patient was diagnosed with CA with atypical clinical features using pathological and immunohistochemical analysis findings.

Mandato et al. reported that approximately 60% of their study patients underwent simple tumor excision, and 38% had positive margins. Further, the patients who underwent tumor excision were followed up for a mean observation period of 46.6 months. They also reported that local recurrence or metastasis was not consistently observed in patients with CA having atypia or sarcomatous transformation [2]. In the case reports of Khmou et al. and Aydin et al., the patients did not experience tumor recurrence more than 6 months after treatment [4, 16]. However, in 2002, McCluggage et al. described the case of a patient who was included in the study by Mandato et al.; the patient underwent adequate excision, but experienced tumor recurrence at 6 months postoperatively, without malignant transformation [2, 10].

This study suggested that CA could recur despite adequate excision, although the tumor might not be invariably malignant. Hormone therapy as an alternative treatment may potentially produce the expected effect of tumor treatment without recurrence. It has been reported that hormone therapy has been used for AA treatment among these similar diseases (CA, AMFB, and AA) [17]. This case study suggested a possible effect of hormone therapy on the expressions of ER and PgR and a susceptible age for CA. However, no case has yet been reported to clarify the abovementioned suggestions. Most case reports involved patients who underwent tumor resection. This study speculated that these patients underwent surgery mostly for CA arising near the body surface.

In current case study, CA was detected in the retroperitoneal space during an initial pregnancy examination. Previous reports of CA during pregnancy are rare. In 2011, Kairdir et al. reported a case of AMFB that arose in the vagina at 14 weeks of gestation. The tumor was initially similar to Bartholin's cyst [2, 8], although its speculated size increased from 3 to 8 cm in the posterolateral space of the vagina. On clinical evaluation, the patient did not have any other abnormal signs, and she underwent complete local tumor excision. Nevertheless, the case report did not contain detailed information about her pregnancy and abnormal findings [18]. In contrast, the patient in current case study had pyelonephritis at 16 weeks of gestation, with an abnormal pregnancy progression. The incidence of acute pyelonephritis during pregnancy was reported to be 1–2% [19]. Furthermore, 20–30% of patients develop uterine contractions due to pyelonephritis, and the rate of premature delivery is 7–8% [20, 21] [22]. Studies reported that nulliparity and second- or third-trimester pregnancy are risk factors of acute pyelonephritis [19–21] [22]. The patient in this case study was nulliparous, and a large tumor was found at the beginning of the pregnancy, similar to the case reported by Cetinkaya et al. [18]. However, in this case study, the uterus was widely displaced superiorly, and urinary tract dysfunction developed due to the large tumor, which might have caused pyelonephritis.

Based on the present findings, we suggest that the timing and plan of treatment should be considered based on the characteristics of the patient and tumor (benign or malignant). It is possible that the tumor location and size can affect adjacent organs. Regarding the surgical methods, we had a joint meeting with the surgical team and discussed the method of approach, the surgical process, and the possible complications. Based on the tumor size and location, the trance vaginal approach may be required; therefore, we decided to perform open surgery to remove the tumor completely, considering the slight possibility of malignancy. However, we might need to discuss the invasiveness, the complication of the surgery, or other treatments based on the individual case, conforming to the goal of treatment. Particularly in the surgical approach, based on the size of the tumor, whether solid or cystic, its location, and the primary organ, it might be possible to discuss other surgical methods, such as laparoscopic or robotic surgery. Notably, the primary organ of the tumor would be one of the crucial points to be discussed when considering the surgical approach. In this case, it was difficult to detect the site of the tumor origin; however, as previously mentioned, a small part of the vagina ruptured during resection due to the closeness of the surface of the tumor to the vagina. Based on this surgical observation, it may be deduced that the origin of the tumor was the mesenchymal tissue around the vagina. Importantly, predicting the origin organ would increase the effectiveness of the subsequent treatment.

The findings of this study suggest that the risk of recurrence or metastasis was underestimated in previous reports. However, recurrence, especially in reproductive-age women, may influence sexual activity or pregnancy, and thus long-term follow-up should be considered.

In conclusion, a case of CA diagnosed during pregnancy was encountered. The diagnosis of CA was difficult due to the rarity of the disease. The patient developed acute pyelonephritis, probably due to urinary tract dysfunction induced by the giant tumor; additionally, the pyelonephritis led to a miscarriage. The efficacy of surgical treatment and pregnancy outcomes were discussed in this study, although the patient's CA was a benign tumor. Further studies are required to determine the efficacy of conservative treatments, such as hormone therapy, in patients with CA.

## Data Availability

Not applicable.

## Abbreviations

CA: Cellular angiofibroma
AA: Aggressive angiomyxoma
AMFB: Angiomyofibroblastoma
MRI: Magnetic resonance imaging
CTRX: Ceftriaxone
αSMA: α Smooth muscle actin
ER: Estrogen receptor
PgR: Progesterone receptor
